# Characterization of Non-O157 STEC Infecting Bacteriophages Isolated from Cattle Faeces in North-West South Africa

**DOI:** 10.3390/microorganisms7120615

**Published:** 2019-11-26

**Authors:** Emmanuel W. Bumunang, Tim A. McAllister, Kim Stanford, Hany Anany, Yan D. Niu, Collins N. Ateba

**Affiliations:** 1 Department of Microbiology, Faculty of Natural and Agricultural Sciences, North-West University, Mafikeng Campus, Private Bag X2046, Mmabatho 2735, South Africa; emmanuel.bumunang@canada.ca; 2 Agriculture and Agri-Food Canada, Lethbridge Research and Development Centre, Lethbridge, AB T1J 4B1, Canada; 3 Alberta Agriculture and Forestry, Lethbridge, AB T1J 4V6, Canada; Kim.Stanford@gov.ab.ca; 4 Agriculture and Agri-Food Canada, Guelph Research and Development Centre, Guelph, ON N1G 5C9, Canada; hany.anany@canada.ca; 5 Department of Ecosystem and Public Health, Faculty of Veterinary Medicine, University of Calgary, Calgary, AB T2N 1N4, Canada

**Keywords:** Shiga toxin-producing *Escherichia coli*, biocontrol, non-O157 *E*. *coli*

## Abstract

Non-O157 Shiga toxin-producing *Escherichia coli* (STEC) *E. coli* are emerging pathotypes that are frequently associated with diseases in humans around the world. The consequences of these serogroups for public health is a concern given the lack of effective prevention and treatment measures. In this study, ten bacteriophages (phages; SA20RB, SA79RD, SA126VB, SA30RD, SA32RD, SA35RD, SA21RB, SA80RD, SA12KD and SA91KD) isolated from cattle faeces collected in the North-West of South Africa were characterized. Activity of these phages against non-O157 STEC isolates served as hosts for these phages. All of the phages except SA80RD displayed lytic against non-O157 *E.* *coli* isolates. Of 22 non-O157 *E*. *coli* isolates, 14 were sensitive to 9 of the 10 phages tested. Phage SA35RD was able to lyse 13 isolates representing a diverse group of non-O157 *E*. *coli* serotypes including a novel O-antigen Shiga toxigenic (wzx-Onovel5:H19) strain. However, non-O157 *E*. *coli* serotypes O76:H34, O99:H9, O129:H23 and O136:H30 were insensitive to all phages. Based on transmission electron microscopy, the non-O157 STEC phages were placed into *Myoviridae* (*n* = 5) and *Siphoviridae* (*n* = 5). Genome of the phage ranged from 44 to 184.3 kb. All but three phages (SA91KD, SA80RD and SA126VB) were insensitive to *EcoRI-HF* and *HindIII* nucleases. This is the first study illustrating that cattle from North-West South Africa harbour phages with lytic potentials that could potentially be exploited for biocontrol against a diverse group of non-O157 STEC isolated from the same region.

## 1. Introduction

Non-O157 Shiga toxin-producing *Escherichia coli* (STEC) are emerging pathotypes that are frequently associated with diseases ranging from diarrhoea to a more complicated haemorrhagic colitis in humans [1]. The most common non-O157 STEC are O26, O45, O103, O111, O121 and O145. These serogroups are called the “big six“ because they are often associated with severe illness and death in humans and have been declared as adulterants by the United States Department of Agriculture [2]. The public impact of non-O157 STEC strains on humans is worsened by an overall lack of effective treatment and prevention measures even for those that are susceptible to antimicrobial agents [3]. Against this background, numerous outbreaks of human infections caused by non-O157 STEC strains have been reported globally [4,5]. Beef and dairy cattle harbor a diverse group of non-O157 STEC [6,7] that can potentially contaminate beef products or water sources used for irrigation. Thus, effective pathogen control measures in ready to eat products are required especially in case of disease outbreaks.

Bacteriophage (phage) therapy has been revisited following the increasing emergence of antimicrobial resistant bacteria [8]. First investigated in the early 20th century [9], phage therapy was largely abandoned by Western countries due to safety and efficacy concerns and the widespread availability of antibiotics [10]. Recent developments such as the successful use of bacteriophage to treat a multidrug-resistant *Acinetobacter baumannii* systemic infection in a patient in California has sparked renewed interest in phage therapy [11,12]. Phages are obligate parasites of bacteria and are considered the most numerous biological entities in nature with an estimated 10^31^ phages on earth [13]. Phages that infect and kill bacteria hosts by lysis are termed lytic or virulent phages while temperate phages can either lyse or lysogenize their host [14]. The lytic potential of phages is being exploited in different areas in the agro-food industry, specifically in the detection of foodborne pathogens [15,16] and as biocontrol agents [17,18].

A *Listeria monocytogenes*-specific phage cocktail, ListShield™, was the first commercial product to be used as a “generally recognized as safe” phage food additive in ready to eat meat and poultry products [19]. This was followed by EcoShield^TM^ which targeted *E*. *coli* O157:H7 in ground beef and SalmoFresh^TM^, targeting *Salmonella typhimurium* [20]. The characterization of phages for potential use either as alternative treatment or in combination with antimicrobials against foodborne pathogens such as STEC, is an on-going process [21,22]. Additionally, studies have characterized *E*. *coli* O157 phages from cattle in the North-West Province, South Africa [23], but this study did not attempt to isolate phages with activity against other serogroups. At the same time, much attention has focused on the big six non-O157-infecting phages globally [24,25,26]. However, as new pathogenic *E. coli* continue to emerge, this study expands on previous investigations by isolating and characterizing phages with lytic activity against diverse serotypes of Shiga toxigenic, non-O157 *E*. *coli* isolated from beef and dairy cattle in South Africa.

## 2. Materials and Methods 

### 2.1. Bacteriophage Isolation, Host Range Determination, Propagation and Titration

Faecal samples were collected from three commercial beef and/or dairy cattle farms in three regions (Rooigrond, Vryburg and Koster) of the North-West Province of South Africa. To isolate phages, samples were enriched as described by Van Twest and Kropinski [27]. Briefly, faeces (3 g) were enriched in 10 mL of 1.7% (*w*/*v*) Tryptic soy broth (TSB) (Difco Laboratories, Detroit, MI, USA) inoculated with 100 µL of overnight bacteria culture isolated from the same faecal samples, incubated at 37 °C with shaking at 170 rpm for 24 h and then centrifuged at 10,000× *g* for 10 min. Supernatant (phage lysates) were filtered through a 0.22 µM syringe filter (C.C Imelmann Ltd., Gauteng, South Africa) and the crude lysates were stored at 4 °C.

For a spot test inoculation assay, 80 individual non-O157 bacterial culture previously isolated [6] from the same faecal sample were used. These cultures were grown in TSB for 24 h at 37 °C and 1 mL of the overnight culture was diluted in 9 mL of sterile TSB. Subsequently, 1 mL of the diluted sample was flooded on 1.5% (*w*/*v*) Tryptic soy agar (TSA) (Difco Laboratories, Detroit, MI, USA) plates. The remaining bacterial inoculum on the plates was aspirated using sterile pipette tips and plates were left for 10 min to dry at room temperature. After which 10 µL of each crude phage lysate was spot inoculated onto the bacterial lawn and incubated at 37 °C for 24 h. Plates were observed for clear lytic zones on the bacterial lawn. Ten crude phage lysates in TSB that showed lytic activity were preserved in triplicates in a 2 mL eppendorf tubes at 4 °C for two months. The bacterial lawn with lytic activity were used as host strains for subsequent analysis of the crude phage lysates. Both non-O157 *E*. *coli* strains and crude phage lysates were transported to the Lethbridge Research and Development Centre, Canada in accordance with Public Health Agency of Canada regulations (https://www.canada.ca/en/public-health/services/laboratory-biosafety-biosecurity/human-pathogens-toxins-act.html, http://www.tc.gc.ca/eng/tdg/page-1296.html).

Crude phage lysates were purified by using three consecutive cycles of plaque purification using the soft agar overlay technique [28]. Then, 100 μL of a mid-log phase phage-bacterial host culture and phage lysate were mixed with 3 mL of molten top agar (0.3% *w/v* agar) and overlaid onto Modified nutrient agar plates (Dalynn Biologicals, Calgary, AB, Canada). One plaque was picked using sterile cut pipette tips and placed in a 1.5 mL eppendorf tubes containing 9 mL of lambda diluent (10 mM Tris-CL, pH 7.5, 8 mM MgSO_4_) for further purification. The lytic capability of the purified phages for 22 non-O157 *E*. *coli* strains (Supplementary Materials Table S1) was tested using the spot-test inoculation technique as described above. These non-O157 *E*. *coli* strains were previously isolated from cattle faecal samples collected from the same three commercial beef and/or dairy farms in three regions (Rooigrond, Vryburg and Koster) of North-West Province. Bacterial strains were previously characterized by PCR and whole genome sequencing (WGS), [6]. Phage stock filtrates were prepared using the host strains as describe by [29]. The titers of phages in the stock filtrate (10^8^–10^9^ PFU/mL) were later determined by the soft agar overlay method [28]. In order to determine the host range, of the phages, 10 µL of purified lysate was pipetted onto a lawn of 22 non-O157 *E*. *coli* strains and incubated at 37 °C for 24 h. Plates were observed for the formation of plaques.

### 2.2. Transmission Electron Microscopy (TEM)

To examine the morphologies of the phages, ultracentrifugation of the phage suspension was performed at 23,000× *g* for 1 h. The supernatant was discarded, and samples were re-suspended in sterile water. Purified phages were deposited on carbon-coated Formvar films on copper grids, stained with 2% uranyl acetate and images were captured using a Field electron and ion (FEI) Tecnai electron microscope (Tecnai G2 F20 model FEI USA) at 200 KV accelerating voltage.

### 2.3. Genome Size Estimation and Restriction Fragment Length Polymorphism

Purified phages (10^8^–10^9^ PFU/mL) were subjected to Pulsed-field gel electrophoresis (PFGE) for estimation of genome size according to the procedure of Lingohr, Frost and Johnson [30] using a Clamped Homogeneous Electric Field-Dynamic Regulation (CHEFDRIII) system (Bio-Rad, Hercules, CA, USA). Briefly, DNase 1 (10 μg mL^−1^) and RNase A (10 μg mL^−1^) (Sigma-Aldrich, Okaville, ON, Canada) were added to 30 mL phage stock crude lysates and incubated at room temperature for 1 h with continuous stirring to digest residual bacterial nucleotides. Phage lysates were later concentrated overnight at 4 °C by adding polyethylene glycol (PEG) 8000 (Sigma-Aldrich, St. Louis, MO, USA) to a final concentration of 10% *w/v* [28]. PFGE analysis was performed using the following conditions: initial time 2.2 s; final time 54.2 s; voltage 6 V, angle: 120° and a run time of 18 h using *Salmonella* Braenderup reference standard (H9812) as a marker. For restriction enzyme digestion analysis, phage DNA embedded in 1% SeaKem Gold agarose (Lonza, Rockland, ME, USA) was digested with *EcoRI-HF* and *HindIII*, for 4 h at 37 °C. The plugs were then subjected to PFGE for 5 h using a pulse time of 1.0–45.0 s, 6 V cm^−1^ alongside a low range PFGE marker (1 kb plus; New England Biolabs). Gels were stained in ethidium bromide for 30 min and images captured on a Gel Doc imaging system (Alpha Innotech, San Leandro, CA, USA).

## 3. Results

### 3.1. Isolation and Morphology of the Phages

A total of 10 phages were isolated from faecal samples collected in the three different regions of South Africa (Table 1). Each phage was assigned a descriptor as described by Kropinski, Prangishvili and Lavigne [31], vB (bacterial virus), followed by Eco (*Escherichia coli*), M or S (*Myoviridae* or *Siphoviridae*), SA (South Africa), numbers (sample identity) followed by KD or VB or RD or RB (sampling region). For example, a phage isolated from the Koster region was designated vB_EcoS_SA12K, with SA12KD as the short form.

**Figure 1:**
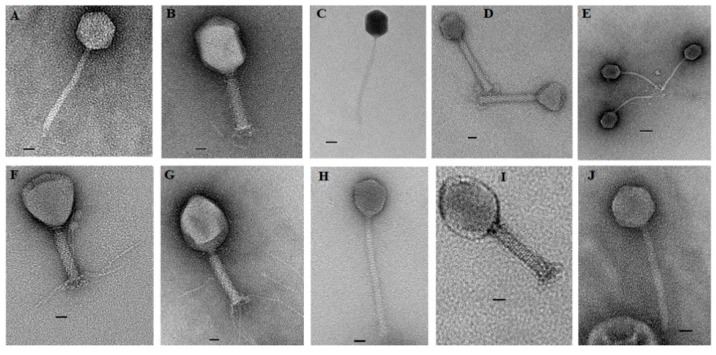
Transmission electron microscopy of 10 non-O157-infecting phages negatively stained with 2% uranyl acetate. (**A**) T1-like phage (SA12KD); (**B**) T4-like phage (SA79RD); (**C**) T1-like phage (SA80RD); (**D**) no genus assigned (SA91KD); (**E**) T1-like phage (SA126VB); (**F**) T4-like phage (SA20RB); (**G**) T4-like phage (SA21RB); (**H**) T1-like phage (SA30RD); (**I**) T4-like phage (SA35RD) and (**J**) T1-like phage (SA32RD). Bar represents 20 nm.

Characterization of phage morphology by transmission electron microscopy identified a wide range of diversity among isolated phages (Figure 1). All phages were members of the order *Caudovirales*. Phages SA79RD, SA35RD, SA20RB, SA21RB and SA91KD had large icosahedral heads with long contractile tails, indicative of the *Myoviridae* [32]. However, phages SA79RD, SA35RD, SA20RB and SA21RB shared similar icosahedral head shape, diameter (82–100 nm by 129–133 nm) and tail length (24–25 nm by 132–134 nm) with visible base plates and tail fibers. These four phages exhibit a morphology similar to T4-like phages. Meanwhile, phage SA91KD had a head and tail with a diameter of 70 by 73 nm and 23 by 154 nm, respectively, but lacked tail fibres and could not be classified using existing criteria within the family *Myoviridae*. In contrast, phages SA12KD, SA80RD, SA126VB, SA30RD and SA32RD had small (50–70 nm by 67–81 nm) icosahedral heads with the long non-contractile tails (9–13 nm by 174–200 nm) indicative of the *Siphoviridae* [32]. These phages were characterized as T1-like phages. Four of the 5 phages (SA12KD, SA126VB, SA30RD and SA32RD) of the *Siphoviridae* family, possessed a long flexible tail with a terminal disk-like structure making them candidates for the proposed subfamily “*Jerseyvirinae*” [33] Table 1.

### 3.2. Host Range

**Figure 2:**
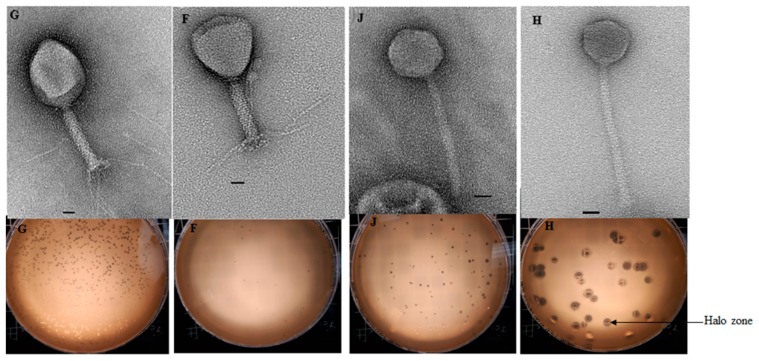
Overlay agar plates of the *Myoviridae* (SA20RB (**F**) and SA21RB (**G**)) and *Siphoviridae* (SA30RD (**H**) and SA32RD (**J**)) phage. Indicating halos around lysis zone illustrating depolymerase activity. Bar represents 20 nm.

All but one phage (SA80RD) showed activity to at least one strain of non-O157 *E*. *coli* (Table 2). However, *E. coli* serotypes O76:H34, O99:H9, O129:H23 and O136:H30 were found to be insensitive to all phages tested. Four phages (SA35RD, SA79RD, SA20RB and SA21RB), had the broadest host range as these phages could lyse non-O157 across serotypes including wzx-Onovel5:H19, O17:H18, O22:H21, wzx-Onovel24:H20, O26:H11, O40:H19, O87:H7 O156:H25, O108:H2, O116:H21, O140:H21, O154:H10 and O163:H19. Serotype wzx-Onovel5:H19 was the most sensitive bacteria since it was lysed by 7 different phages. *Myoviridae* (SA20RB and SA21RB) and *Siphoviridae* (SA30RD and SA32RD) phages exhibited halo zones around the plaques with larger plaque sizes being associated with *Siphoviridae* phages than *Myoviridae* phages (Figure 2).

### 3.3. Phage Genome Size and Restriction Fragment Length Polymorphisms Analysis

**Figure 3:**
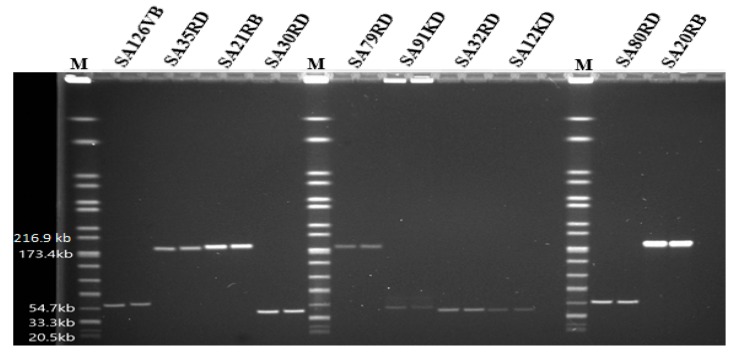
Pulsed-field gel electrophoresis analysis of phages indicating approximate whole genome sizes with lanes in duplicates, run with a *Salmonella* Braenderup reference standard (H9812) marker (M).

**Figure 4:**
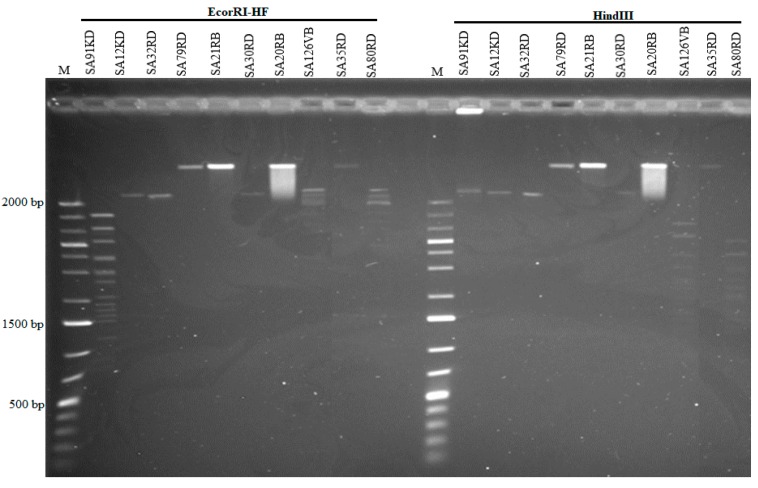
Restriction analysis of phages using *EcoRI-HF* and *HindIII* and a low range marker (M) 1 kb plus.

Based on PFGE, the genome size of phages ranged from 44 to 184.3 kb (Figure 3). Some phages shared a common genome size of about 184.3 kb (SA35RD, SA21RB, SA79RD and SA20RB), 44 kb (SA30RD, SA32RD and SA12KD), with phage SA91KD, SA80RD and SA126VB having genome sizes of about 47.5, 54.7 and 60.5 kb, respectively. Genomic DNA from phage (SA80RD) of the *Siphoviridae* family was cleaved by *EcoRI-HF* and *HindIII*, while SA126VB was only cleaved by *HindIII*. In contrast, only to *EcoRI-HF* restricted DNA from *Myoviridae* phage (SA91KD) (Figure 4).

## 4. Discussion

This is the first study to isolate and characterize phages with potential activity against a diverse group of non-O157 STEC isolated from South Africa. All *E. coli* and phages were isolated from cattle faeces collected in the North-West Province of South Africa. Phages isolated in this study possessed either a long flexible tail or double layer contractile tail with spikes and belonged to the order *Caudovirales* and family *Siphoviridae* or *Myoviridae* as described by Ackermann [32]. Similar to the present study, phages targeting the “big six” non-O157 *E. coli* (O26, O45, O103, O111, O121, O145) were also members of the *Siphoviridae* and *Myoviridae* families and were isolated from cattle faeces in Canada [24], the USA [25] and from water samples in the USA [26]. As well as the recent report of Korf, Meier-Kolthoff, Adriaenssens, Kropinski, Nimtz, Rohde, van Raaij and Wittmann [34], which indicates a diverse group of myoviruses and siphoviruses from various sources such as surface water, manure sewage and animal faeces with lytic capability for different non-O157 *E*. *coli* from human origin.

Although phage were classified into *Siphoviridae* and *Myoviridae* families, two phages (SA30RD and SA32RD) of the family *Siphoviridae* showed a long flexible tail with a terminal disk-like structure while two (SA12KD and SA126VB) possessed club-shaped spikes and may belong to the proposed genus “*K1glikevirus*” of the subfamily “*Jerseyvirinae*” [33]. Besides being isolated in Canada [33], Jerseyvirnae-like phages have been isolated from swine lagoon effluent in England [35], chicken by-products in South Korea [36], sewage in the USA [37], and humans with diarrhea in Bangladesh [38]. These phages are known to be strictly lytic [39] and thus are potentially good candidates for the control of STEC. The presence of T4 phages (SA79RD, SA35RD, SA20RB and SA21RB) and Jersey-like phages in cattle faecal samples from the North-West South indicate that these phages with lytic activity are good candidates for biocontrol of diverse pathogenic non-O157 *E*. *coli* strains.

A halo surrounding the lysis zone is commonly associated with phages in the order *Caudovirales* [40]. The halos result from enzymes (depolymerase) encoded by phages that have the ability to degrade exopolysaccharide substances (EPS) on the surface of bacterial cells or embedded within biofilms [41]. Anti-biofilm effects on *E*. *coli* [42], *Staphylococcus epidermidis* and *Staphylococcus aureus* [43] have been associated with depolymerase activity as these enzymes can facilitate phage absorption and infection by degrading the EPS in biofilms. In this study, two phages each from the family *Myoviridae* (SA20RB and SA21RB) and *Siphoviridae* (SA30RD and SA32RD) showed depolymerase activity. These phages were capable of lysis of multidrug resistant (O154:H10) and novel (wzx-Onovel5:H19) serotypes indicating that they could potentially control these non-O157 *E*. *coli* pathotypes. Other than a single-dose application of phage as biocontrol agents, combined treatment with antimicrobials is promising [44] as a depolymerase-secreting phage can degrade the ESP exposing cells to antimicrobials. This could be exploited using the depolymerase-secreting phages obtained in this study.

Phage particle size (genome, capsid and tail size) is a contributing factor to plaque size as smaller phages can migrate or diffuse faster after lysis as compared to larger phages [45,46]. The larger plaque size observed with the depolymerase-secreting phages SA30RD and SA32RD compared to SA20RB and SA21RB in this study is in agreement with the finding of a previous study of *Siphoviridae* phages from cattle faeces which found that they generated larger plaque sizes [47]. Furthermore, this observation is in line with the above contributing plaque factor as phage SA30RD and SA32RD possessed a smaller genome (about 44 kb) and capsid (67 nm) size compared with larger genome (about 184.3 kb) and capsid (100 and 107 nm) of the SA20RB and SA21RB phage.

Phages that display broad lytic spectra either resist host strains defense mechanisms or infect members of the same species or bacteria which have common targets (receptors) such as pili, teichoic acid, flagella, lipopolysaccharides and outer membrane proteins [48]. The phages with highest lytic spectra in this study belonged to the *Myoviridae* family and were capable of lysing 13/22 (phage SA35RD), 12/22 (phage SA79RD) and 10/22 (phage SA20RB and SA21RB) non-O157 *E*. *coli*. Compared to *Siphoviridae,* others have found that *Myoviridae* phages possess a broader activity against non-O157 *E*. *coli* in Canada [24] and the USA [47]. Also the recent studies of Korf, Meier-Kolthoff, Adriaenssens, Kropinski, Nimtz, Rohde, van Raaij and Wittmann [34] revealed that myoviruses lysed 39.1% of *E*. *coli* tested compared to 17.2% for siphoviruses. Coliphages unlike other phages, do not preferentially bind to bacterial proteinaceous or polysaccharide receptors [49], thus the ability to recognize a range of receptors might contribute to the broader lytic ability of *Myoviridae* as the *Siphoviridae* associate with a single receptor [48]. While broad-spectrum phages are preferred candidates for biocontrol as they can overcome mutant or resistant strains [50], narrow-spectrum phages could be used to target a specific host or to be used synergistically with other phages. This raises the possibility that the SA35RD and SA32RD, narrow spectrum infecting-phages (wzx-Onovel5:H19, O102:H4 and O17:H18 serotypes) could be used synergistically with broad spectrum non-depolymerase producing phage(s) to yield a much broader lytic spectrum.

Restriction of phage DNA to prevent the expression of phage early proteins is one of the defense mechanisms used by bacteria to overcome phage infection [51]. Phages have also developed methods to evade degradation in the host cell by either lacking enzymatic restriction sites or modifying restriction sites so that they are no longer susceptible to cleavage [52]. All but one phage (SA91KD) from the *Myoviridae* family were resistant to *EcoRI-HF* and *HindIII*, as SA91KD was susceptible to *EcoRI-HF* only. A similar lack of restriction sites for these enzymes (*EcoRI-HF* and *HindIII*) has been reported in other studies of non-O157 *E*. *coli* T4-like infecting-phages [24] and an O157 T4-like infecting phage from water samples in Iran [53]. The resistance to restriction enzymes might be due to the absence or modification of the restriction site to make it unavailable for digestion by specific enzymes. In T4 phages, beta-glycosyltransferase is an enzyme responsible for DNA methylation, which aids in the glycosylation process of a methylated hydroxymethylcytosines (HMC) found in dsDNA [54]. Some bacterial restriction systems can bind to hydroxymethylcytosine-containing DNA, preventing infection by phages having HMC [51]. A glycosylated T4 DNA, a product of methylcytosine blocks the binding of specific restriction enzymes [51,55] and might have been responsible for insensitivity of Myoviruses (SA79RD, SA20RB, SA35RD and SA21RB) to restriction enzymes in this study. Similarly, Siphoviruses (SA12KD, SA30RD and SA32RD) were also insensitive to *EcoRI-HF* and *HindIII*, while phage SA80RD was sensitive to *EcoRI-HF* and *HindIII* and SA126VB to *HindIII* only. The sensitivity of these two phages to the restriction enzymes can be attributed to a lack of restriction sites, as glycosylated DNA defence mechanisms are not commonly associated with T-odd phages [56]. The ability to resist these digestion enzymes also makes these phages potential agents for phage-based biocontrol.

## 5. Conclusions

Phages are potential alternatives to antimicrobials as they are natural killers of bacteria. Non-O157 *E*. *coli* phages have been given little attention in South Africa, and this study isolated diverse phages from cattle faeces collected in the North-West Province of South Africa that can lyse STEC and antimicrobial resistant strains with biofilm-forming ability. The heterogeneous nature of these phages, the lytic spectra and ability to secret depolymerase enzymes and to resist restriction digestion enzyme *EcoRI-HF* and *HindIII* activity makes some of these phages good candidates for biocontrol. Whole genome sequencing of these phages to determine a lack of virulence factors is the next step in their evaluation as biological control agents.

## Figures and Tables

**Table 1:** Taxonomy and dimensions of 10 non-O157 *E*. *coli* phages.

Sampling Area	Phage ID	Family	Subfamily	Head Dimension (nm)		Tail Dimension (nm)	
				Width	Height	Width	Height
Rooigrond dairy	SA79RD	*Myoviridae*	-	100 ± 0	133 ± 0	24 ± 2	133 ± 0
Koster dairy	SA91KD	*Myoviridae*	-	70 ± 3	73 ± 4	23 ± 0	154 ± 4
Rooigrond beef	SA20RB	*Myoviridae*	-	100 ± 0	129 ± 4	25 ± 2	134 ± 2
Rooigrond beef	SA21RB	*Myoviridae*	-	82 ± 2	129 ± 4	25 ± 4	132 ± 2
Rooigrond dairy	SA35RD	*Myoviridae*	-	95 ± 7	133 ± 2	24 ± 2	133 ± 2
Rooigrond dairy	SA80RD	*Siphoviridae*	-	50 ± 6	67 ± 0	9 ± 2	200 ± 0
Koster dairy	SA12KD	*Siphoviridae*	“*Jerseyvirinae*”	70 ± 0	78 ± 2	13 ± 2	197 ± 0
Vryburg beef	SA126VB	*Siphoviridae*	“*Jerseyvirinae*”	67 ± 7	81 ± 4	12 ± 2	191 ± 2
Rooigrond dairy	SA30RD	*Siphoviridae*	“*Jerseyvirinae*”	67 ± 0	67 ± 0	10 ± 0	200 ± 3
Rooigrond dairy	SA32RD	*Siphoviridae*	“*Jerseyvirinae*”	67 ± 0	69 ± 2	10 ± 0	174 ± 2

**Table 2:** Lytic ability of 10 non-O57 *E*. *coli* phages.

Serogroup	Bacteriophage									
	SA20RB	SA79RD	SA126VB	SA30RD	SA32RD	SA35RD	SA21RB	SA80RD	SA12KD	SA91KD
O156:H25	+	+	-	-	-	+	+	-	-	+
O108:H2	+	+	+	-	-	+	+	-	-	+
O136:H30	-	-	-	-	-	-	-	-	-	-
O99:H9	-	-	-	-	-	-	-	-	-	-
wzx-Onovel24:H20	+	+	-	-	-	+	+	-	-	-
O140:H21	+	-	-	-	-	+	+	-	-	-
O102:H4	-	-	+	-	-	-	-	-	-	-
O129:H23	-	-	-	-	-	-	-	-	-	-
O17:H18	-	+	-	-	+	+	-	+	-	-
O76:H34	-	-	-	-	-	-	-	-	-	-
O26:H11	-	+	-	-	-	+	-	-	-	-
O129:H23	-	-	-	-	-	-	-	-	-	-
O154:H10	+	-	-	-	-	-	+	-	-	-
O116:H21	-	+	-	-	-	+	+	-	-	-
wzx-Onovel5:H19	+	+	+	+	-	+	+	-	-	+
O87:H7	+	+	-	-	-	+	+	-	-	-
O129:H21	-	+	-	-	-	+	+	-	-	-
O26:H11	-	+	-	-	-	+	-	-	-	-
O26:H11	-	-	-	-	-	-	+	-	-	-
O163:H19	+	+	+	+	-	+	-	-	-	+
O40:H19	+	+	-	-	-	+	-	-	-	-
O22:H21	+	-	-	-	-	-	-	-	-	-

+ = lytic activity; - = no lytic activity.

## Supplementary Materials

Supplementary materials can be found at https://www.mdpi.com/2076-2607/7/12/615/s1.
