# Supplementary material for: Characterization of Non-O157 STEC Infecting Bacteriophages Isolated from Cattle Faeces in North-West South Africa
Source: Microorganisms. 2019 Nov 26;7(12):615. doi: 10.3390/microorganisms7120615 (PMC6956337; doi:10.3390/microorganisms7120615)
Supplement: Supplementary file 1 [file microorganisms-07-00615-s001.pdf]

Table S1: Sequence annotation results for non-O157 *E. coli* serotype determinants (O- and H-antigen sequences) and Shiga toxins (Stx) types

Sampling Region	Isolate Number	O-Type	H-Type	Stx Types Based on PCR Detection	Stx Types Based on Annotated Results
Koster dairy	11	O156	H25	1	1
	12	O108	H2	1&2	none
	14	O136	H30	1	none
	15	O99	H9	2	none
Vryburg beef	22	wzx-Onovel24	H20	none	none
	25	O140	H21	1&2	none
	30	O102	H4	2	none
Rooigrond dairy	32	O129	H23	2	none
	37	O17	H18	2	2
	38	O76	H34	2	none
	42	O26	H11	1&2	none
	50	O129	H23	2	none
	69	O26	H11	2	none
	72	O26	H11	2	none
	76	O163	H19	2	none
	77	O40	H19	2	2
	80	O22	H21	none	none
Rooigrond beef	56	O154	H10	1&2	none
	60	O116	H21	2	2
	64	wzx-Onovel5	H19	1&2	2
	67	O87	H7	1&2	none
	68	O129	H21	1&2	2

None: no *stx* gene detected
